# Application of Pb Isotopes and REY Patterns in Tracing Heavy Metals in Farmland Soils from the Upper-Middle Area of Yangtze River

**DOI:** 10.3390/ijerph20020966

**Published:** 2023-01-05

**Authors:** Yongqiang Ning, Bizheng Yang, Shaochen Yang, Jiaxin Ye, Junjie Li, Limin Ren, Zhifu Liu, Xiangyang Bi, Jinling Liu

**Affiliations:** 1 Hubei Key Laboratory of Critical Zone Evolution, School of Earth Sciences, China University of Geosciences, Wuhan 430074, China; 2 State Key Laboratory of Ore Deposit Geochemistry, Institute of Geochemistry, Chinese Academy of Sciences, Guiyang 550002, China; 3 University of Chinese Academy of Sciences, Beijing 100049, China

**Keywords:** agricultural soil, heavy metals, source apportionment, farmland type

## Abstract

Farmland heavy metal pollution—caused by both human activity and natural processes—is a major global issue. In the current study, principal component analysis (PCA), cluster analysis (CA), rare earth elements and yttrium (REY) analysis, and isotope fingerprinting were combined to identify sources of heavy metal pollution in soil from different farmland types in the upper-middle area of the Yangtze River. The concentrations of Zn and Cu were found to be higher in the vegetable base and tea plantation soil compared with their concentrations in the orangery soil. On the other hand, greater accumulation of Cd and Pb was observed in the orangery soil versus the vegetable base and tea plantation soils. Influenced by the type of bedrock, REY was significantly enriched in the orangery soil and depleted in the vegetable base soil, as compared with the tea plantation soil. The Pb isotopic compositions of the tea plantation (1.173–1.193 for ^206^Pb/^207^Pb and 2.070–2.110 for ^208^Pb/^206^Pb) and vegetable base (1.181–1.217 for ^206^Pb/^207^Pb and 2.052–2.116 for ^208^Pb/^206^Pb) soils were comparable to those of coal combustion soil. The compositions of ^206^Pb/^207^Pb (1.149–1.170) and ^208^Pb/^206^Pb (2.121–2.143) in the orangery soil fell between those observed in soils obtained from coal combustion and ore smelting sites. Using the IsoSource model, the atmospheric Pb contributions of the vegetable base, tea plantation, and orangery soils were calculated to be 66.6%, 90.1%, and 82.0%, respectively, and the bedrock contributions of Pb were calculated to be 33.3%, 9.90%, and 18.1%, respectively. Based on the PCA, CA, and REY results, as well as the Pb isotope model, it appears that heavy metals in the orangery soil may be derived from atmospheric deposition and bedrock weathering, while heavy metals in the vegetable base and tea plantation soils may be derived from mining and the use of fertilizer.

## 1. Introduction

China is one of the world’s largest miners and consumers of metals [1]. The improper treatment and discharge of heavy metals continue to cause severe environmental pollution to the soil, water, and air. Based on the results of China’s national soil pollution survey, the country’s farmland soil pollution area extends beyond 10^7^ ha (8.30% of cultivated area) [2,3]. Heavy metals are the primary contamination in this soil, including cadmium (Cd), copper (Cu), chromium (Cr), and lead (Pb) [2]. The pollution of Chinese farmland soils with heavy metals is on the rise [3]. Crops (12.0 × 10^6^ t) grown in these regions are also polluted with heavy metals [4]. Furthermore, these heavy metals can remain in the soil for decades and continue to harm human health [4]. Hence, it is necessary to control heavy metals emissions and reduce the heavy metals pollution of agricultural soil in China.

Human activities and natural factors influence the accumulation of heavy metals in agricultural soils [2,5,6]. Important natural factors, such as the type of bedrock, soil properties, and topography, influence the accumulation of heavy metals in soil [5]. The geochemistry of the bedrock determines the overall concentration of these metals in the soil [7]. For example, soils derived from mafic and ultrabasic rocks contain higher levels of metals compared with soils derived from siliceous rocks [8]. The status of heavy metals in soil also differs due to topographic differences [9]. Topography affects the process of weathering and pedological processes, which, in turn, directly affect the accumulation of heavy metals in soil [5,10]. Rezapour et al. (2014) [9] examined soils from northern Iran and found a greater weathering rate of metal-bearing rocks in the soil on the northern slope (which also accumulated more metals) compared with the soil on the southern slope. Clay minerals, calcium carbonate, organic matter, and iron oxides, all formed during the weathering and pedological processes of parent material, and have significant impacts on the translocation and accumulation of metals [5]. Similarly, Hseu et al. (2015) [11] found that the weathering of serpentine in the parent material promotes the accumulation of Cr and nickel (Ni) in soil. 

The accumulation of heavy metals in soil is also influenced by anthropogenic factors such as farming practices and crop types [6]. In northern China, paddy fields accumulate more heavy metals than wheat fields due to differences in irrigation practices [12]. Fertilizer overuse and short crop rotation cycles have also been found to increase Cd accumulation in vegetable fields in northwest China (up to 1.5 times more than in arable soils) [13]. The application of zinc (Zn) fertilizer is an important factor affecting the accumulation of Zn in soil and crops during the cultivation of zinc-loving crops (such as tea) [14]. Moreover, the widespread use of pesticides to improve the yields of leafy and vegetable crops contributes to the accumulation of heavy metals in soil [15]. Heavy metal pollution arising from different types of farming has been a concern for several decades [6]. However, the total amount of heavy metals discharged in different regions varies, and the cumulative amounts of heavy metals in soils differs. Therefore, it is difficult to assess whether the heavy metal pollution in different regions is caused by differences in agricultural methods or industrial emissions. To successfully minimize heavy metals contamination in soils, it is necessary to trace the sources of heavy metals across various agricultural methods in the same region.

At present, various methods are employed for quantitative source apportionment, including principal component analysis (PCA), cluster analysis (CA), rare earth elements and yttrium (REY) analysis, and Pb isotopic fingerprinting [2,6,16]. The composition of REY (La, Ce, Pr, Nd, Sm, Eu, Gd, Tb, Dy, Y, Ho, Er, Tm, Yb, Lu, and Y) and Pb isotopes varies across different sources, such as parent materials, coal combustion, and ores smelting [2,17,18]. Hence, REY and Pb isotopes have been used widely to investigate the origins of pollution [18,19], for trace provenance [2,20], and to understand environmental processes [21]. However, the Pb isotopes of different sources are too similar to be distinguishable [18] and REY is predominantly used to identify the source of bedrock in geological processes [19]. Compared with Pb isotopes, REY, PCA, and CA can be used to identify heavy metals from different sources but cannot provide direct proof of the origin of heavy metals. Hence, the combined application of Pb isotopes, REY, PCA, and CA may support the identifications of the sources of heavy metals. This would be useful toward classifying the sources of heavy metals in the soil, both from human activities and natural processes.

Zigui City is an important producer of tea, citrus, and vegetables in the upper-middle area of Yangtze River. As Zigui City was founded more recently, there are fewer heavy metals in the soil due to historical emissions, as compared to soils from big cities with long histories. The current study analyzed soil samples from several farmland types (i.e., tea plantation, vegetable base, and orangery) in Zigui City to ascertain the respective heavy metal pollution levels. Further, the effects of farming practices, crop types, bedrock, and atmospheric deposition (coal combustion and ore smelting) were analyzed using PCA, CA, isotope fingerprinting, and REY composition analysis. The results of this study may contribute to the formulation of protection strategies for different farmland types.

## 2. Materials and Methods

### 2.1. Study Area and Sample Collection

**Figure 1:**
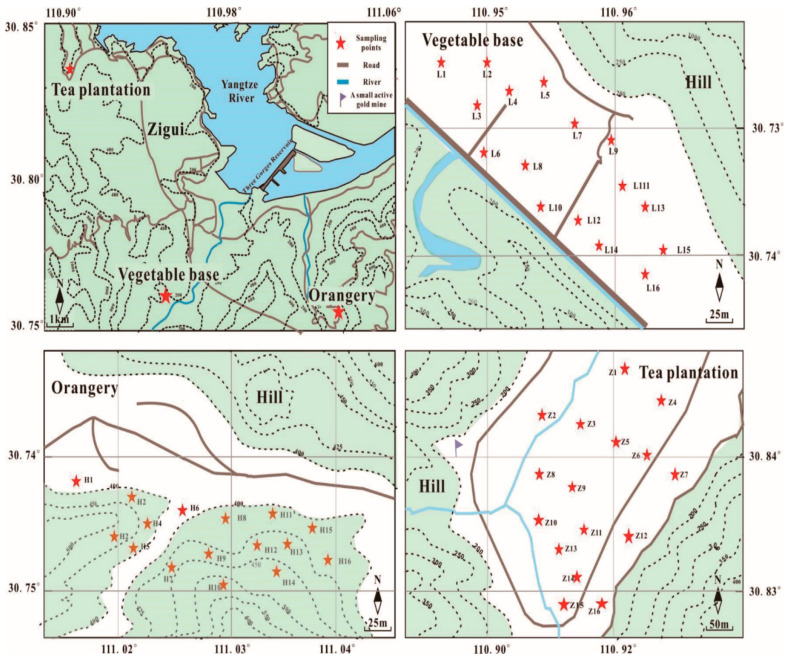
Map of sampling sites in Zigui, the Three Gorges Reservoir region (TGR).

The farmland examined in this study (E 110°18′–110°0′, N 110°38′–31°11′) is located in the area of the upper-middle Yangtze River—Three Gorges Reservoir (TGR); it varies in altitude between 600 and 2057 m (Figure 1). The region has a monsoon-influenced subtropical climate with a mean annual temperature of 17 °C and mean annual precipitation of 1181 mm. The local economy is based on agricultural practices, as well as the light and service industries. Carbonate rocks exist extensively in this region, and magmatic rock and metamorphic rock outcrops occur sporadically in the TGR [22].

Soil samples for the current study (5–15 cm, n = 75) were collected from an orangery (Huajipo orangery, HJP; n = 25), a vegetable base (Zhangjiachong vegetable base, ZJC; n = 24), and a tea plantation (Lanlinxi tea plantation, LLX; n = 26) in July 2020 (Figure 1). The bedrocks of the tea plantation, vegetable base, and orangery comprise gray–green pebbled silty sandstone of Nantuo (Nh_3n_), dolomitic and calcareous shale of Doushantuo (Z_1_d^3^), and porphyritic granodiorite (πγδPt_3_), respectively [23,24]. The soil obtained from the tea plantation is a coarse yellow sandy soil belonging to the acid coarse bone soil subclass. The gravel content of the soil is more than 30% and the soil is a yellowish-brown color. The vegetable base soil is gray–purple clay field soil belonging to the retention paddy soil subclass. The sand content in the soil is more than 50%, and the soil is a purple color. The orangery soil is a coarse red sand soil belonging to the acid coarse bone soil subclass. The soil contains 39–50% gravel and 70–80% coarse sand and is a light-yellow color. 

The soil samples were freeze-dried and thoroughly mixed before being pulverized and passed through a 100-mesh sieve prior to analysis. Aliquots of the samples were taken, and the pH was determined after mixing 1 g of soil with 2.5 mL of water [25].

### 2.2. Chemical Analysis

The soil samples (50 mg) were digested in covered Teflon beakers with 1 mL HNO_3_ (65% *v*/*v*) and 1 mL HF (40% *v*/*v*) at 190℃ for 48 h [2]. Next, the digest was evaporated to virtual dryness before being digested with 1 mL HNO_3_ (65% *v*/*v*) and 0.5 mL HClO_4_ (70% *v*/*v*) at 150 °C for 8 h [2]. After cooling, the digestate was diluted to 50 mL with HNO_3_ (3% *v*/*v*) and an Agilent 7900 (Agilent Technologies, Santa Clara, CA, USA) was used to determine the metal concentrations (REY, Pb, Zn, Cr, Cu, and Cd) of the digest. Soil reference materials (GBW07423, Chinese National Standard Soils), parallel samples, and reagent blanks were analyzed to ensure the analytical quality. The recoveries of GBW07423 were 90–104%. The relative standard deviations (RSDs) of the parallel samples were below 7%.

The Pb isotopic compositions of the soil samples were determined using an Agilent 7900. NIST SRM 981 (Pb isotopic standard) was used for sample calibration and analytical control. The RSDs (10 replicates) were found to be under approximately 0.4%. The ^206^Pb/^207^Pb and ^208^Pb/^206^Pb ratios of NIST SRM 981 were 1.0930 ± 0.0020 and 2.1702 ± 0.0045, respectively; these values are close to the SRM 981 reference values.

### 2.3. Rare Earth Elements and Yttrium (REY) Methods

To trace the different bedrocks, the REY concentrations of the samples in the current research were normalized based on the upper continental crust (UCC) [26]. δEu − ∑REY plots and associated regression curves have successfully been used to trace soil provenance in previous studies [16,27]. ∑REY is the sum of the La, Ce, Pr, Nd, Sm, Eu, Gd, Tb, Dy, Y, Ho, Er, Tm, Yb, Lu, and Y concentration. The following equation was used to calculate the *δEu* [27]:

δEu=Eu/Sm×Gd12

where *Eu* is the concertation of Europium in the soil (mg kg^−1^), *Sm* is the Samarium concentration of the soil (mg kg^−1^), and *Gd* is the Gadolinium concentration of the soil (mg kg^−1^).

### 2.4. Source Apportionment of Soil Using Stable Pb Isotopes

The IsoSource model, developed by the Environmental Protection Agency (US), is a method that can be used to quantitatively assess source contributions [28]. In the current study, this model was used with the fractional increment set to 1% and the mass balance tolerance set to 0.02% using the data from ^206^Pb/^207^Pb and ^208^Pb/^206^Pb. This was done to obtain the proportional contributions of the potential sources (including the Pb-Zn ore, coal, diesel, and gasoline with unleaded and natural sources) from the three farmland types. 

### 2.5. Statistical Analysis

Nonparametric tests (Kruskal–Wallis) were used to evaluate differences in median values. The CA were processed on the REY and heavy metals concentration using Origin 2019b, as well as the average connection method and the Euclidean coefficient as the criterion, to form clusters for the analysis. PCA was used to extract factors based on the REY and heavy metals concentration, and a correlation matrix was used to identify the correlations between the different elements.

## 3. Results and Discussion

### 3.1. Distribution of Heavy Metals in Different Farmland Soil Types

The concentrations of heavy metals in the three types of farmland examined in this study are summarized in Table 1 and Table S1. The mean Pb concentration in the soil samples from the orangery (21.4 mg kg^−1^) was higher than those of the tea plantation (15.6 mg kg^−1^) and vegetable base (20.3 mg kg^−1^). The high concentration of Pb in the orangery soil may be due to atmospheric deposition and the parent material of the soil. First, traffic emissions, coal combustion, and mining activities are the most important sources of Pb [18]. Additionally, previous studies have shown that ore smelting and coal combustion are significant sources of heavy metals in Zigui [29]. Moreover, tree leaves absorb heavy metals from the atmosphere [30]; thus, falling leaves may affect the Pb concentration of the soil. In contrast, in vegetable-growing soils, most vegetable leaves do not reach the soil as they are instead consumed by humans. This may lead to low Pb concentrations. Thus, in summary, falling leaves at the orangery may have contributed to the increased Pb level in the orangery soil compared to the vegetable base soil. Additionally, the source of the parent material determines the background value of heavy metals in soil [9]. Granite is the parent material in the orangery, and granite accumulates high levels of Pb. Further, minerals from granite, such as augite and hornblende, are rich sources of Pb [9]. The bedrocks of the vegetable base and tea plantation soil are calcareous and shale sandstone, respectively, and these sedimentary rocks have low levels of Pb. Additionally, sandstone has the lowest levels of Cr, Cu, Zn, and Pb among all sedimentary rocks [31]. The low concentration of Pb in the tea plantation soil compared with those of the orangery and vegetable base soils are consistent with the above.

The mean Cd concentrations and pH of the soil samples from the orangery (0.46 mg kg^−1^ and 6, respectively) and vegetable base (0.42 mg kg^−1^ and 5.7, respectively) were higher than those of the tea plantation (0.31 mg kg^−1^ and 4.7, respectively). Soil acidification may be the key factor affecting the distribution of Cd in the soil from different types of farmland. Additionally, Cd is more easily released from soil at a low pH compared with other heavy metals (e.g., Pb and Cu) due to its lower binding energy [32]. Crop types can also affect the heavy metal content of soil through the activity of root exudates and other root activities [33]. Caffeic acid, flavonoids, and flavins may cause soil acidification and increase heavy metal activity [34,35,36]. Tea plants are typically acid-loving, and their roots can secrete organic acids such as citric acid, succinic acid, oxalic acid, and malic acid to promote the absorption of nutrients by the plants. This can enhance Cd release into the environment [37]. In addition, sandstone is known to have a higher Cd content compared with other types of rocks (e.g., magmatic rock and shale) [38]. Hence, tea planting may significantly affect the Cd content of soil.

The Zn concentration of the orangery soil (60.5 mg kg^−1^) was much lower than those of the tea plantation (80.2 mg kg^−1^) and vegetable base (85.3 mg kg^−1^) soils. The high concentrations of Zn in the tea plantation and vegetable base soils could be associated with agricultural activities. Zn can enhance plant stress resistance, increase seed weight, and change the seed-to-stem ratio [39]. As an antimicrobial agent and growth promoter, Zn and its compounds are often used as fertilizers in agriculture, and this results in Zn penetrating agricultural soil [2]. Fertilizers are one of the main contributors to Zn in soils [2]. The Zn concentration in fertilizers can be as high as 136 mg kg^−1^ [17]. The excessive use of fertilizers on the vegetable base and tea plantation soils probably resulted in the higher concentrations of Zn [17]. The vegetable base and tea plantation were found to use twice as much fertilizer as the orangery.

The concentrations of Cu (43.0 mg kg^−1^) and Cr (63.9 mg kg^−1^) in the tea plantation soil were higher than those of the orangery (22.5 and 38.4 mg kg^−1^, respectively) and vegetable base (39.2 and 44.1 mg kg^−1^, respectively) soils. The tea plantation is in the immediate vicinity of an active gold mine. Mining activities are highly likely to have impacted the soil’s Cu and Cr concentrations because Cu and Cr are significant elements in gold ore. Previous studies have shown that mining activity is a significant factor affecting the concentrations of Cu and Cr in soil from the TGR [18,29].

While different crops can absorb heavy metals from the soil to different extents, previous studies have shown that heavy metals absorption by vegetables, tea, and orange trees is not a key factor affecting heavy metal concentrations in soil [40,41,42]. Therefore, farming practices (e.g., fertilization), bedrock types, and the atmospheric absorption of plants, which differ as a function of farmland type, lead to differences in heavy metal concentrations in the soil.

### 3.2. Rare Earth Elements and Yttrium (REY)

**Figure 2:**
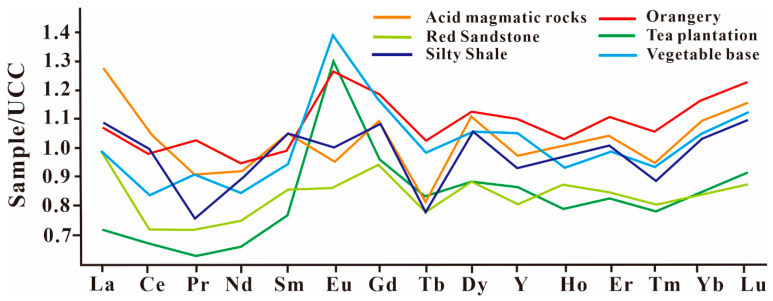
Upper continental crust (UCC)-normalized curves of REY.

**Figure 3:**
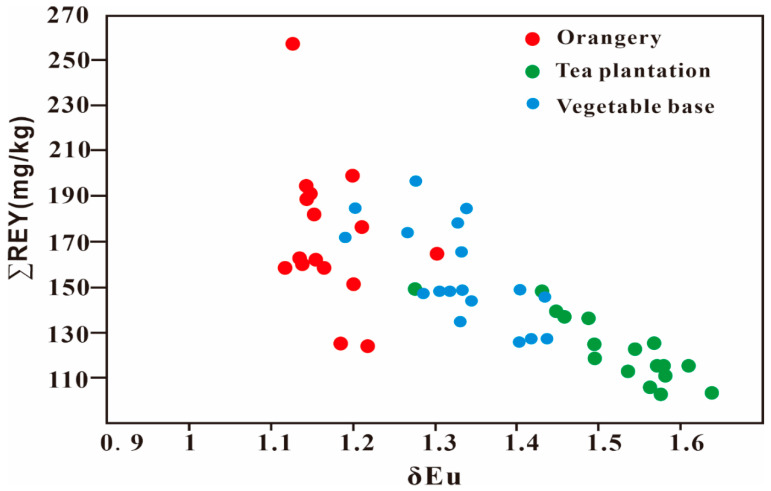
The δEu − ∑REY plot of three types of soils of farmland.

In order to clarify differences in the parent materials, δEu − ∑REY plots and upper continental crust (UCC)-normalized REY curves were drawn for the average soil values of the three different farmland types (Figure 2 and Figure 3). The patterns of the orangery, tea plantation, and vegetable base soils were found to be fundamentally different (Figure 2 and Figure 3). The tea plantation soil showed a negative anomaly of REY, except for Eu; the orangery soil showed positive enrichment of REY while REY in the vegetable base soil varied between the orangery and tea plantation soils (Figure 2). These results suggest different sources of REY. Previous studies have revealed clear differences in REY produced by different parent rocks [43]. The UCC-normalized curves of REY based on the average soil weathering value of different bedrocks in China are summarized in Table S2. The characteristics of REY in the tea plantation soil were more like those of red sandstone (Figure 2). Additionally, the vegetable base soil and purplish red silty shale soil patterns were almost identical (Figure 2). The distribution of REY in orangery soil was consistent with that of soil whose parent rock is acidic magmatic rock (Figure 2). These results are consistent with the parent rock types of the tea plantation, orangery, and vegetable base. Further, the degree of weathering of the different parent rocks can also lead to differences in the distribution of rare earth elements (REEs) in soil [44]. Yttrium (Y), which is always preferentially lost during weathering, has an unusually low affinity for iron (Fe; hydroxyl compounds) oxides compared with Ho [44]. In this study, the Y/Ho ratio was used to determine the degree of weathering of the soil across the different farmland types. The Y/Ho ratios of the tea plantation (30.1 ± 0.791), vegetable base (30.9 ± 0.550), and orangery (29.4 ± 1.36) were almost identical, indicating little difference in the degree of weathering of the soil parent rocks across the three farmland types. Therefore, the patterns of REY in the different farmland soil types serve as an important means of identifying the natural heavy metals sources of the tea plantation, vegetable base, and orangery. Accordingly, it appears that differences in the bedrock may be a significant factor affecting the heavy metal concentrations of the soils.

### 3.3. Pb Isotopes in Soils from Different Farmland Types

**Figure 4:**
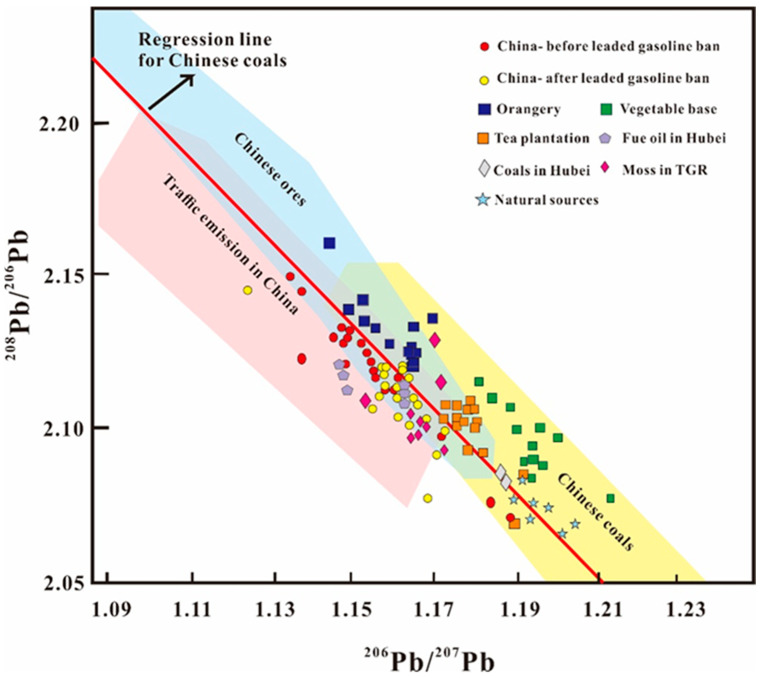
Lead isotope ratios (^206^Pb/^207^Pb vs. ^208^Pb/^207^Pb) in the soil samples. The regression line for Chinese coals was from Bi et al. (2017) [47]; unleaded gasoline and diesel exhausts and local coals (Hubei) from Bi et al. (2017) [47]; mosses from TGR from Liu et al. (2018) [18]; natural backgrounds were from [45,46,48,49].

The isotopic ratios of Pb in the soils varied from 1.149 to 1.217 for ^206^Pb/^207^Pb and from 2.052 to 2.143 for ^208^Pb/^206^Pb. The isotopic ratios differed significantly from those found in nature (1.194 to 1.206 for ^206^Pb/^207^Pb and 2.066 to 2.076 for ^208^Pb/^206^Pb) [18,45,46]. This suggests obvious anthropogenic effects on the soils (Figure 4). The Pb isotopic composition results suggest that Pb concentrations in the samples are influenced by Chinese ores (1.181–1.182 for ^206^Pb/^207^Pb and 2.099–2.101 for ^208^Pb/^206^Pb), coal (1.020–1.183 for ^206^Pb/^207^Pb and 2.088–2.309 for ^208^Pb/^206^Pb), traffic emissions (1.147–1.162 for ^206^Pb/^207^Pb and 2.106–2.126 for ^208^Pb/^206^Pb), and natural sources (1.1373–1.193 for ^206^Pb/^207^Pb and 1.802–2.085 for ^208^Pb/^206^Pb) [47]. Pb is an important component of the TGR, formed through coal combustion and ore smelting [18]. The orangery soil had isotopic compositions of 1.149–1.170 for ^206^Pb/^207^Pb and 2.121–2.143 for ^208^Pb/^206^Pb. The Pb isotopic compositions in the soil samples from the vegetable base were in the range of 1.181–1.217 for ^206^Pb/^207^Pb and 2.052–2.116 for ^208^Pb/^206^Pb. The tea plantation soil had Pb isotopic ratios of 1.173–1.193 for ^206^Pb/^207^Pb and 2.070–2.110 for ^208^Pb/^206^Pb. In comparison to the vegetable base results, the Pb isotopic compositions in the orangery and tea plantation soils were shifted toward Chinese ore and coal combustion sources, which indicates that ore smelting and coal combustion significantly contributed to the Pb concentration of the orangery and tea plantation soils (Figure 4). In comparison to the tea plantation and orangery, the Pb isotopic composition of the vegetable base soil was shifted toward natural sources (Figure 4). This indicates a more pronounced contribution of natural sources in the vegetable base soil. The high Pb isotopic compositions of the three farmland types were close to those of coal combustion and Chinese ore sources. This suggests that coal combustion and ore smelting are the dominant contributors to Pb pollution. It is worth noting that mosses obtained from the Zigui country (1.154–1.173 for ^206^Pb/^207^Pb and 2.094–2.129 for ^208^Pb/^206^Pb) and sediments from the TGR region (1.165–1.185 for ^206^Pb/^207^Pb and 2.084–2.114 for ^208^Pb/^206^Pb) have similar Pb isotopic signatures to those in the current study [18]. Together, these results suggest that coal combustion and ore smelting are the most significant sources of Pb in the TGR region.

**Figure 5:**
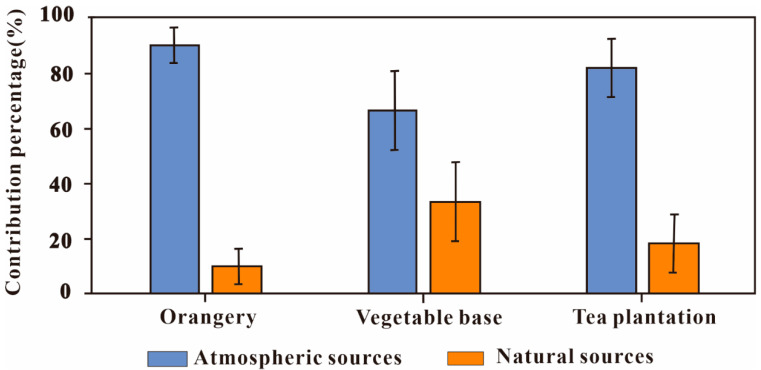
The proportional contributions of different sources of heavy metal to the soils from the three types of farmland.

To quantitatively assess the relative contributions of potential sources using the IsoSource model for the different farmland types, two endmembers were defined as follows: atmospheric sources (including Pb-Zn ore, coal, diesel, and unleaded gasoline) and natural sources (uncontaminated soil) [47]. For this calculation, the individual endmembers were represented by the mean Pb isotope values. IsoSource software was used to estimate the contributions of Pb from atmospheric and natural sources. The atmospheric contributions to the tea plantation (83.3–96.9%) and orangery (71.2–92.6%) soils were higher than that of the vegetable base soil (52.1–81.3%). Further, the natural contributions of Pb to the tea plantation (3.10–16.7%) and orangery (7.4–28.8%) soils were lower than that for the vegetable base soil (18.7–47.9%; Figure 5). These results indicate that the orangery and tea plantation soils accumulated more atmospheric Pb than the vegetable base soil. Previous studies have shown that plant leaves can absorb Pb from the atmosphere [30]. Further, evergreen tea and orange trees can accumulate more Pb from coal combustion than vegetables [50]. The falling leaves of tea trees and orange trees may affect the composition of Pb isotopes in the soil. Vegetable leaves do not usually enter the soil and are instead consumed by humans. This affects the natural source characteristics of Pb isotopes in vegetable base soils. In addition, more frequent irrigation and loosening of the soil during crop rotations can promote the loss of Pb from soil [51]. Compared with the orangery and tea plantation soils, the vegetable base soil underwent more frequent crop rotation (3 or 4 times per year^−1^). This might have contributed to the loss of Pb from atmospheric sources (coal combustion and ore smelting) on the soil surface. 

The high ore smelting Pb contribution (52.1% ± 10.6%) in the tea plantation soil should also be noted (Table S3). Nearby mining activities in the area are highly likely to have impacted the isotopic composition of the tea plantation soil. This is in line with the high concentration of Cu in the soil from the tea plantation.

### 3.4. Source Apportionment of Heavy Metals in the Soil of Different Farmland Types

**Figure 6:**
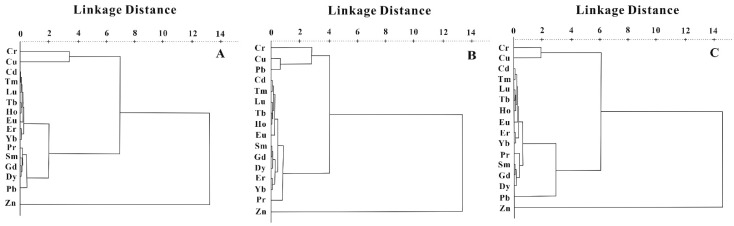
Hierarchical dendrograms for REY and heavy metals in soils from the tea plantation (**A**), vegetable base (**B**), and orangery (**C**).

Nonparametric ANOVA revealed significant differences (*p* < 0.01) in the heavy metals concentration of three farmland types. The sources of the heavy metals in the soils from the three farmland types were further classified according to CA (Figure 6). For the tea plantation and orangery soils, four clusters were identified: (1) Zn, (2) Cd and REY, (3) Cr and Cu, and (4) Pb. In the vegetable base soil, three clusters were identified: (1) Zn, (2) Cd and REY, and (3) Pb, Cr, and Cu. The long distance of Zn in cluster 1, observed across the three farmland types, suggests that Zn may have the same input source. As mentioned above, Zn is likely to enter agricultural soil via fertilization. CA showed a strong correlation between Cd and REY in cluster 2 across the different farmland types, indicating a common source of these elements. The REY patterns exhibited lithogenic-element characteristics. The source of Cd across the three farmland types was bedrock. Further, Cr and Cu were also correlated in the orangery, tea plantation, and vegetable base soils, suggesting another common source. Previous studies have shown that Cu and Cr are likely to be emitted from gold mining in the TGR [18,29]. Gold mining could be a significant source of Cu and Cr for the three farmland types. Cluster 4 predominantly contained Pb in the tea plantation and orangery soils. The observed Pb isotopic compositions indicate that Pb was predominantly generated through coal combustion and ore smelting. This cluster may point to atmospheric sources. It is also worth mentioning that cluster 3 for the vegetable base soil contained Pb, Cr, and Cu. The results of the Pb isotope contribution model revealed that atmospheric Pb accounted for more than 50% of the vegetable base soil. This cluster indicates that the vegetable base soil accumulated more Pb from the local gold mine compared with that released by coal combustion and gasoline.

To further classify the metals of different sources, PCA was performed (Table S4). The PCA results revealed three factors (tea plantation), three factors (vegetable base), and two factors (orangery) of metals, respectively, accounting for 87.8%, 88.9%, and 82.8% of the total variance, respectively. Factor 1 explained 64.4% (tea plantation), 66.6% (vegetable base), and 71.6% (orangery) of the total variance, respectively, and was characterized by positive factor loadings of REYs. The average positive loading of heavy metals in the orangery soil (0.192) was higher than that of the vegetable base (0.101) and tea plantation (0.0526) soils. These results indicate that, compared with the tea plantation and vegetable base soils, heavy metals in the orangery soil were more affected by bedrock. Factor 2 for the tea plantation and orangery soils explained 15.6% and 11.2% of the total variance, respectively, while factor 3 for the vegetable base soil explained 10.1% of the total variance. These factors were characterized by positive factor loadings for Pb and Cd. These results indicate that the contents of Pb and Cd in the soils from the different types of farmland were affected by atmospheric sources. Moreover, factor 2 for the vegetable base soil explained 12.3% of the total variance while factor 3 for the tea plantation soil explained 7.76% of the total variance. These factors were characterized by positive factor loadings for Zn. These results indicate that, compared with the orangery soil, the Zn concentrations of the tea plantation soil and vegetable base soil were more affected by fertilization.

The CA and PCA results revealed the sources of heavy metals in the vegetable base, orangery, and tea plantation soils. However, the accumulation of heavy metals across the different farmland types differed. Compared with the orangery soil, the Zn and Cu concentrations were higher in the vegetable base and tea plantation soils. Greater accumulation of Cd and Pb was observed in the orangery soil as compared to the vegetable base and tea plantation soils. Thus, taking the PCA, CA, REY patterns, and Pb isotope model results together, it can be concluded that the heavy metals found in the orangery soil may result from atmospheric deposition and bedrock weathering. In contrast, the heavy metals content of the vegetable base and tea plantation soils may result from mining activities and fertilizer use.

## 4. Conclusions

As shown in the current study, heavy metals contribute significantly to the pollution of farmland. The heavy metals content of soil cultivated with short-cycle crops (e.g., vegetables) is susceptible to fertilizer application, while the heavy metals content of soil cultivated with long-term crops (e.g., orange trees, tea) is susceptible to atmospheric subsidence and weathering of bedrock. Given the different contents and sources of heavy metals in different types of farmland soils, future studies should pay more attention to identifying the sources and accumulation of heavy metals in different kinds of crops in order to minimize the accumulation of heavy metals in the human body due to the consumption of different types of crops.

## Figures and Tables

**Table 1:** Mean concentrations (mean ± 1 SD) of heavy metals and pH (mean ± 1 SD) in soils from the orangery, vegetable base, and tea plantation.

Location	Cu (mg kg^−1^)	Pb (mg kg^−1^)	Cd (mg kg^−1^)	Zn (mg kg^−1^)	Cr (mg kg^−1^)	pH
Tea plantation	43.0 ± 6.81	15.6 ± 4.15	0.31 ± 0.0618	80.2 ± 24.2	63.9 ± 16.5	4.70 ± 0.50
Orangery	22.5 ± 4.34	21.4 ± 2.23	0.46 ± 0.0558	60.5 ± 9.57	38.4 ± 8.39	6.00 ± 0.40
Vegetable base	39.2 ± 11.8	20.3 ± 4.24	0.42 ± 0.0939	85.3 ± 26.4	44.1 ± 7.10	5.70 ± 0.40
Average	34.9	19.1	0.40	75.3	48.8	5.47

## Data Availability

The data presented in this study are available on request from the corresponding author.

## Supplementary Materials

The following supporting information can be downloaded at: https://www.mdpi.com/article/10.3390/ijerph20020966/s1, Table S1. Concentrations of heavy metal in the soils from the three types of farmlands (mg/kg). Table S2. The mean fractionation value of REY in soils derived from different parent materials normalized to the UCC (upper continental crust). Table S3. Contribution of different sources to Pb in soils of the three types of farmlands. Table S4. The principal component analysis (PCA) loadings of heavy metals and REY in soil from the three types of farmlands.
