# Supplementary material for: Application of Pb Isotopes and REY Patterns in Tracing Heavy Metals in Farmland Soils from the Upper-Middle Area of Yangtze River
Source: Int J Environ Res Public Health. 2023 Jan 5;20(2):966. doi: 10.3390/ijerph20020966 (PMC9858800; doi:10.3390/ijerph20020966)
Supplement: Supplementary file 1 [file ijerph-20-00966-s001.zip › ijerph-2093170-supplementary.pdf]

This supporting material contains 4 tables (Table S1-S4)

Table S1. Concentrations of heavy metal in the soils from the three types of farmlands (mg/kg).

	Tea plantation					Orangery					Vegetable base				
	Cr	Cu	Zn	Cd	Pb	Cr	Cu	Zn	Cd	Pb	Cr	Cu	Zn	Cd	Pb
1	69.05	43.46	106.53	0.33	12.91	29.86	22.26	89.62	0.49	19.39	42.15	54.10	110.47	0.34	17.97
2	67.36	43.10	101.61	0.26	16.38	27.45	17.92	70.69	0.43	19.28	39.92	41.70	99.23	0.48	18.28
3	67.59	57.38	111.55	0.40	15.86	41.91	26.81	94.66	0.53	26.87	40.52	47.68	95.17	0.31	15.51
4	82.40	56.52	115.92	0.32	16.68	37.52	23.88	84.62	0.46	22.28	40.61	41.11	92.19	0.45	19.62
5	64.09	59.60	116.99	0.35	15.59	27.43	14.02	82.68	0.45	20.14	45.87	45.24	104.83	0.49	21.04
6	93.65	37.29	90.69	0.15	6.62	41.54	24.09	92.97	0.47	22.86	48.11	77.57	156.01	0.55	19.16
7	54.97	47.12	106.25	0.32	16.62	32.33	21.01	94.21	0.57	23.64	66.93	37.79	184.91	0.31	18.56
8	61.17	40.52	102.48	0.33	16.38	43.23	23.87	99.91	0.50	22.05	47.03	47.56	137.15	0.55	30.73
9	42.55	40.33	116.63	0.43	27.64	44.35	19.10	80.39	0.36	18.85	37.53	34.95	86.88	0.36	18.70
10	62.43	43.89	199.95	0.32	14.21	26.39	21.61	87.76	0.41	18.75	45.33	27.55	93.78	0.52	25.88
11	61.71	43.45	127.90	0.32	14.13	48.78	25.19	103.90	0.48	22.32	46.81	29.94	97.34	0.54	26.06
12	98.06	36.94	108.19	0.32	19.28	52.16	26.43	106.67	0.54	21.74	51.75	36.88	105.30	0.37	21.11
13	90.13	42.18	110.35	0.25	13.14	50.77	29.32	106.67	0.50	24.93	55.36	34.43	107.03	0.62	24.04
14	50.50	39.84	90.17	0.25	16.79	44.42	33.09	100.07	0.54	20.79	40.53	31.28	84.76	0.44	20.65
15	43.27	36.93	90.37	0.37	15.49	46.99	23.87	90.93	0.51	19.57	38.61	27.72	75.09	0.38	16.27
16	54.24	41.03	102.50	0.30	12.32	43.64	24.43	92.68	0.39	20.69	43.07	30.95	96.47	0.41	25.30
17	46.82	45.34	100.07	0.33	20.26						37.89	34.08	86.84	0.30	12.89
18											48.66	48.32	98.87	0.39	19.43

Table S2. The mean fractionation value of REY in soils derived from different parent materials normalized to the UCC (upper continental crust).

	La	Ce	Pr	Nd	Sm	Eu	Gd	Tb	Dy	Y	Ho	Er	Tm	Yb	Lu
Acid magmatic rock	1.28	1.05	0.91	0.92	1.05	0.95	1.09	0.81	1.11	0.97	1.00	1.04	0.94	1.09	1.16
Neutral magmatic rock	1.20	1.00	0.77	0.85	0.92	0.97	0.96	0.73	0.99	0.88	0.94	0.85	0.92	0.97	1.03
Basic magmatic rock	1.47	1.13	1.18	1.18	1.35	1.25	1.49	1.20	1.43	1.25	1.31	1.28	1.19	1.27	1.34
Eruptive rock	1.24	1.08	0.85	0.86	0.94	0.85	0.88	0.58	0.90	0.79	0.78	0.87	0.81	0.98	1.03
Shale	1.28	1.11	0.94	1.00	1.13	1.14	1.18	0.95	1.18	1.05	1.08	1.08	0.97	1.11	1.13
Sandstone	1.37	1.06	1.00	1.05	1.20	1.19	1.26	0.97	1.17	1.04	1.06	1.10	1.00	1.10	1.13
Limestone	1.41	1.15	1.03	1.12	1.28	1.30	1.34	1.08	1.32	1.17	1.21	1.24	1.11	1.21	1.25
Red sandstone	0.99	0.72	0.72	0.75	0.86	0.86	0.94	0.78	0.90	0.81	0.88	0.85	0.81	0.85	0.88
Sandy shale	1.09	1.00	0.75	0.90	1.06	1.00	1.08	0.78	1.06	0.93	0.96	1.01	0.89	1.03	1.09
Fluvial sedimentary rocks	1.19	0.96	0.92	0.97	1.11	1.14	1.18	0.94	1.14	1.00	1.04	1.05	0.97	1.05	1.06
Lacustrine sedimentary rocks	1.28	0.94	0.83	0.93	1.08	1.02	1.15	0.72	1.10	0.95	0.98	0.98	0.92	0.97	1.00
Marine sedimentary rocks	0.46	0.23	1.09	0.34	0.36	0.50	1.35	0.38	1.30	0.42	1.14	1.19	1.11	0.44	0.41
Anemoarenyte	1.04	0.74	0.77	0.74	0.82	0.91	0.89	0.66	0.85	0.76	0.86	0.79	0.94	0.83	0.88
Tillite	1.18	0.94	0.83	0.92	1.06	1.01	1.17	0.95	1.11	0.95	1.05	1.00	0.94	0.98	1.03

Data from China Environmental Monitoring Station. Background values of soil elements in China. Beijing: China Environmental Science Press, 1990.180-215

Table S3. Contribution of different sources to Pb in soils of the three types of farmlands.

	Ore Smelting	Natural source	Coal combustion	Gasoline
Tea plantation	52.1±10.6%	9.9±6.8%	23.8±12.6%	14.2±10.8%
Vegetable base	15.8±11.5%	33.3±14.6%	33.6±22.9%	17.2±12.9%
Orangery	25.0±14.4%	18.1±10.7%	34.7±20.8%	22.3±16.0%

Table S4 The principal component analysis (PCA) loadings of heavy metals and REY in soil from the three types of farmlands

	Tea plantation			Vegetable base			Orangery	
	PC1(64.4%)	PC2(15.6%)	PC3(7.76%)	PC1(66.6%)	PC2(12.3%)	PC3(10.0%)	PC1(71.6%)	PC2(11.2%)
Cr	0.103	-0.466	0.194	0.075	0.565	0.178	0.237	0.141
Cu	0.117	0.166	0.554	0.068	0.395	-0.339	0.226	0.313
Zn	-0.008	0.149	0.756	0.028	0.698	0.047	0.257	0.298
Cd	0.070	0.593	0.072	0.162	0.036	0.493	0.123	0.565
Pb	-0.002	0.559	-0.233	0.173	0.029	0.606	0.117	0.545
Pr	0.281	0.217	-0.094	0.266	-0.157	0.234	0.260	0.004
Sm	0.314	0.044	-0.075	0.297	-0.072	0.080	0.288	-0.057
Eu	0.300	-0.131	0.044	0.285	0.028	-0.036	0.288	0.002
Gd	0.319	-0.011	-0.040	0.298	-0.050	0.100	0.290	-0.076
Tb	0.320	-0.022	-0.064	0.303	-0.039	-0.020	0.299	-0.115
Dy	0.320	-0.029	-0.022	0.297	-0.001	-0.072	0.294	-0.169
Ho	0.318	-0.038	-0.008	0.300	-0.008	-0.121	0.288	-0.140
Er	0.317	-0.024	0.016	0.296	-0.002	-0.143	0.277	-0.195
Tm	0.308	-0.001	-0.032	0.287	-0.025	-0.216	0.266	-0.175
Yb	0.316	-0.020	0.001	0.291	0.009	-0.171	0.271	-0.195
